# The importance of ultrasound-guided biopsy: lesson from a case of liver metastasis from uveal melanoma

**DOI:** 10.1007/s40477-024-00909-5

**Published:** 2024-06-12

**Authors:** Maria Boe, Susanna Vicari, Andrea Boccatonda, Fabio Piscaglia

**Affiliations:** 1 Division of Internal Medicine, Hepatobiliary and Immunoallergic Diseases, IRCCS Azienda Ospedaliero-Universitaria di Bologna, Bologna, Italy; 2 Internal Medicine, Bentivoglio Hospital, AUSL Bologna, Via Marconi N 35 Bentivoglio, 40010 Bologna, Italy; 3 Department of Medical and Surgical Sciences (DIMEC), University of Bologna, Bologna, Italy

**Keywords:** Melanoma, Liver, Ultrasound, CEUS, Metastasis

## Abstract

Melanoma is an extremely aggressive malignant neoplasm. Uveal melanoma is the most common primary intraocular malignancy in adults, representing 3–5% of all melanomas. Liver metastases can be clinically detected in 10–20% of patients with metastatic disease from cutaneous melanoma. However, while liver is typically not the first site of disease spread in cutaneous melanoma, ocular melanoma has been showed to primarily metastasize from the eye to the liver; indeed, liver metastases are detected in approximately 87% of patients with metastatic uveal melanoma. Therefore, liver metastasis can be challenging to identify in early stages, thus being essentially asymptomatic until the disease has advanced. Here we report the case of a patient who came to our ultrasound unit reporting a large liver mass. Both contrast-enhanced abdominal computed tomography and magnetic resonance imaging did not establish a definitive diagnosis. The final diagnosis was made only through an ultrasound-guided biopsy of the mass, thus revealing a uveal melanoma metastasis. This is followed by a review of the literature on imaging follow-up of patients with melanoma.

## Introduction

Melanoma is an extremely aggressive malignant neoplasm arising from melanocytes, that are specialized cells involved in the production of the melanin. The majority of melanocytes are located in the skin, but they can also be found and develop in other anatomical sites, such as the uvea [1]. Indeed, uveal melanoma is the most common primary intraocular malignancy in adults, representing 3–5% of all melanomas [2, 3]. Liver metastases can be clinically detected in patients with metastatic disease from uveal melanoma [2–4]. The long-term surveillance of patients with melanoma, and in particular with uveal melanoma, is a topic of debate. In contrast to conjunctival melanoma, uveal melanomas are known to metastasize hematogenous, and a common explanation is the lack of lymphatics within the eye [2–4]. Here we report the case of a patient with gross liver involvement by a mass that cannot be well characterized by using traditional methods such as computed tomography and magnetic resonance. Contrast enhanced ultrasound will highlight a particular perfusional feature of the mass, and it will serve as a guide to perform the biopsy procedure that will lead to the final diagnosis.

## Case report

A 68-year-old woman was referred to gastroenterological consultation reporting elevated levels of transaminases and lactate dehydrogenase on blood exams. Her past medical history was characterized by a previous diagnosis of ocular melanoma in 2020 treated with brachytherapy, currently under oncological follow-up. A second-level imaging method was required. Therefore, the patient underwent a complete abdominal computed tomography (CT) scan with contrast medium.

**Fig. 1:**
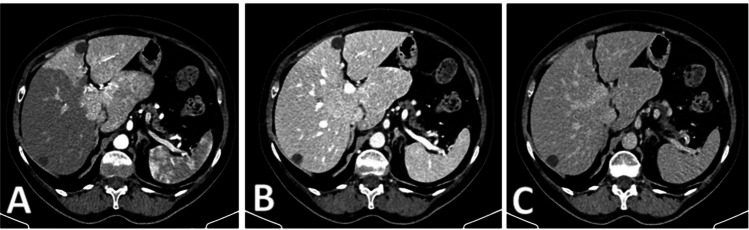
Abdominal Computed tomography with contrast medium: there is an enlarged liver with a pathological mass involving the entire caudate lobe and parts of the adjacent right segments, measuring up to 10 cm in maximum diameter. On contrast medium, it was characterized by inhomogeneous contrast enhancement (**A**), thus appearing slightly hypodense in the venous and late phases (**B** and **C**), with multiple markedly hypodense areas within it. The mass exerted significant compression on the hepatic veins at their confluence with the inferior vena cava, leading to hypodense appearance in the right hepatic lobe during the arterial phase due to altered blood supply. Multiple small hypodense lesions of up to 7 mm in size were observed in the remaining liver parenchyma

The CT scan (Fig. 1) demonstrated the presence of an enlarged liver with a pathological mass involving the entire caudate lobe and parts of the adjacent right segments, measuring up to 10 cm in maximum diameter. It exhibited inhomogeneous contrast enhancement, thus appearing slightly hypodense in the venous and late phases, with multiple markedly hypodense areas within it. The mass exerted significant compression on the hepatic veins at their confluence with the inferior vena cava, leading to hypodense appearance in the right hepatic lobe during the arterial phase due to altered blood supply. Moreover, several small hypodense lesions of up to 7 mm in size were observed in the other segments of liver parenchyma. The findings were considered of uncertain interpretation, and it was recommended to promptly conduct further diagnostic assessment through MRI with liver specific contrast medium.

**Fig. 2:**
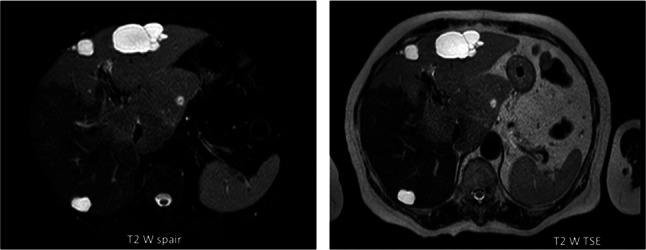
Magnetic Resonance Imaging: the liver is markedly enlarged at the level of the caudate lobe. Three hepatic areas can be found: 1. a central one of approximately 8.5 × 5.5 × 7.0 cm which occupies part of the caudate lobe and the pericaval region: this region is characterized by signal hyperintensity in T1-weighted sequences with some gaps of greater contextual hyperintensity, appears slightly hypointense in the T2-weighted sequences (even the contextual gaps), takes on the contrast medium (while the gaps described above appear markedly hyperintense). This area has an expansive effect and compresses the adjacent structures, in particular the inferior vena cava and the outlet of the hepatic veins. 2. the remaining portions of the left liver (II, III and IV segments) appears diffusely hyperintense in the arterial phase, while it is hypointense in the equilibrium and hepatobiliary phases; in the context of this area, hyperintense millimeter nodules can be found in T1-weighted sequences, unrecognizable in T2-weighted sequences and hyperintense in contrast-enhanced sequences. 3. the right lobe presents contrastographic characteristics typical of a normal liver. According to radiological report, those findings were suggestive for an expansive lesion (lymphoma? FNH like lesion?) of 8.5 × 5.5 cm in the caudate lobe—pericaval region with contextual hemorrhagic areolae and millimetric satellite nodules in the left lobe

MRI with liver specific contrast medium (Fig. 2) confirmed the presence of a voluminous pathological area in the caudate lobe, characterized by signal hyperintensity in T1-weighted sequences and slight hypointensity in T2-weighted sequences. The lesion showed contrast enhancement, and there were some markedly hyperintense gaps within it. The MRI confirmed that the mass had an expansive effect, leading to the compression of the inferior vena cava and the hepatic veins. The other portions of the left liver appeared diffusely hyperintense in the arterial phase and hypointense in the hepatobiliary phases. Within this context, millimetric hyperintense nodules were observed in the T1-weighted sequences, not recognizable in the T2-weighted sequences and hyperintense in the sequences with contrast agent. The right lobe showed contrast features consistent with a healthy liver.

Therefore, the second level imaging method did not clarify the nature of the lesion, by raising suspicions of either a focal nodular hyperplasia (FNH)-like lesion or lymphoma. Subsequently, the patient was referred to our internal medicine ultrasound unit to perform a contrast-enhanced ultrasound (CEUS) and subsequent liver biopsy.

**Fig. 3:**
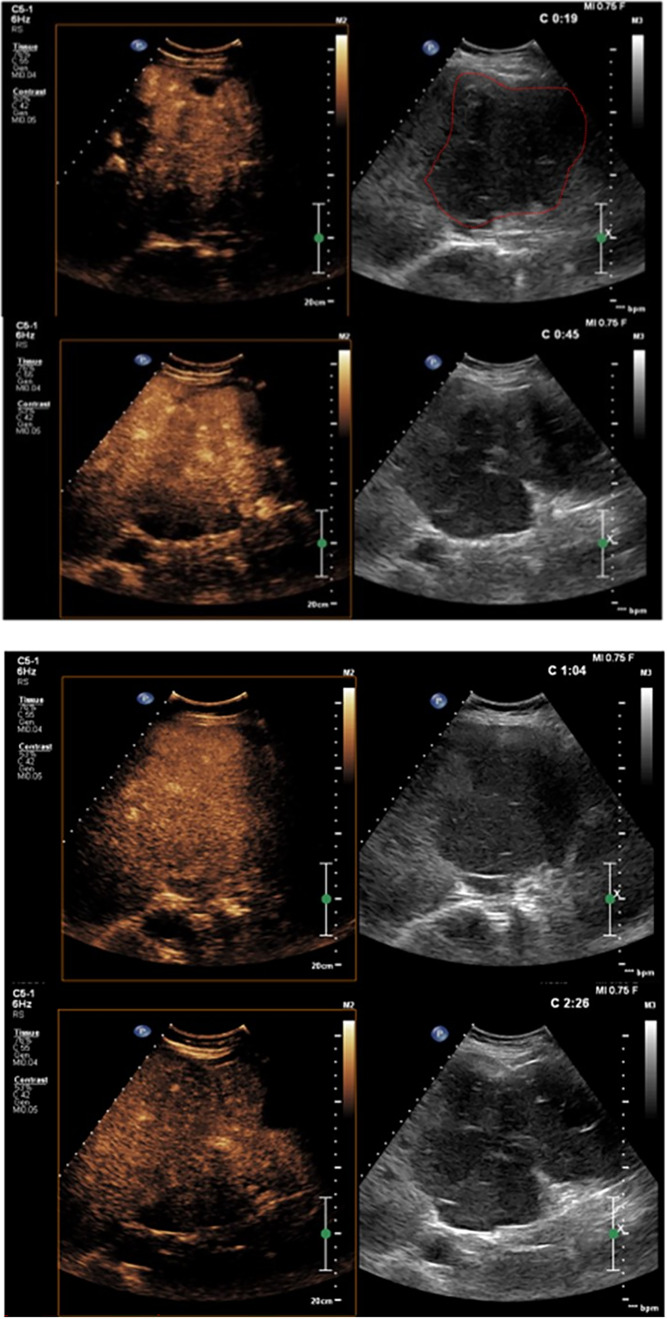

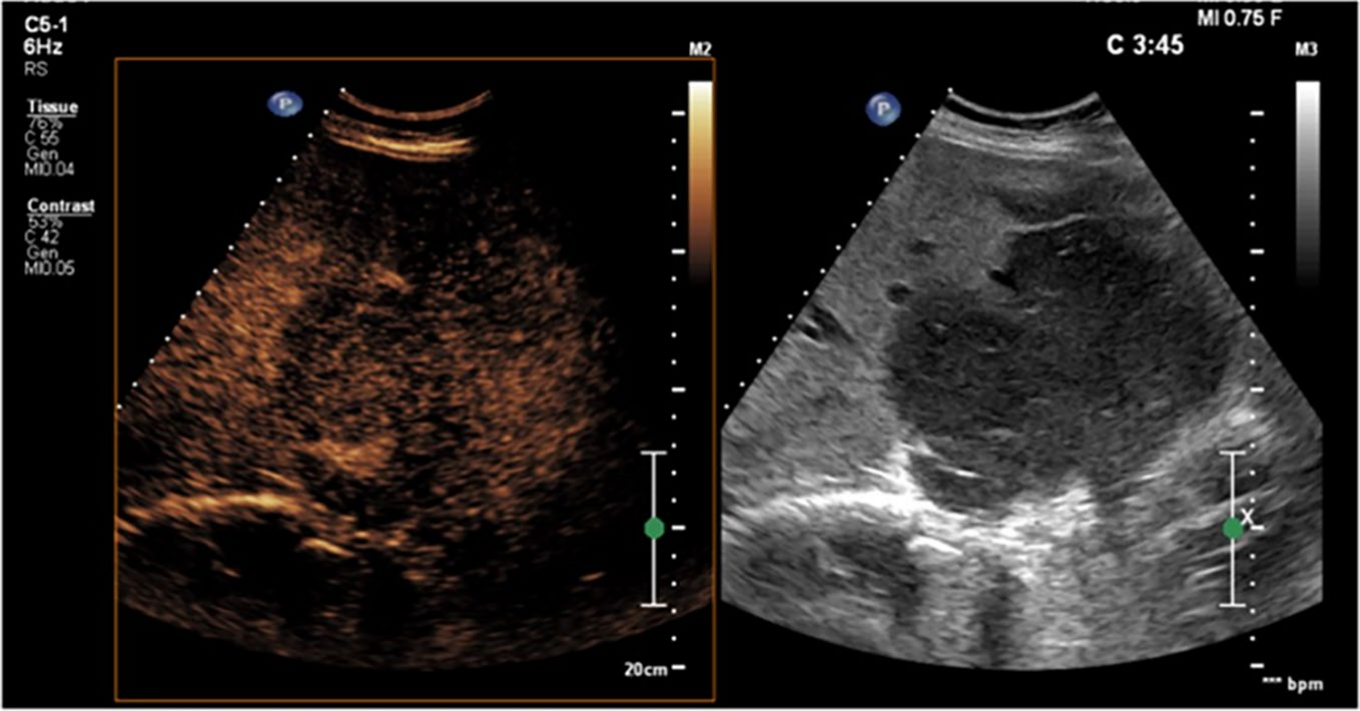
CEUS examination focused on the liver. The red dotted line outlines the hypoechoic lesion on B-mode examination. In the arterial phase, a clear hyperenhancement of the pathological area of the liver is evident compared to the normal one prevailing on the right segments. In the portal phase, isoenhancement is evident. The pathological area presents a certain degree of wash-out compared to the normal parenchyma, albeit not complete, in the late phase, best assessed after 3 min

**Fig. 4:**
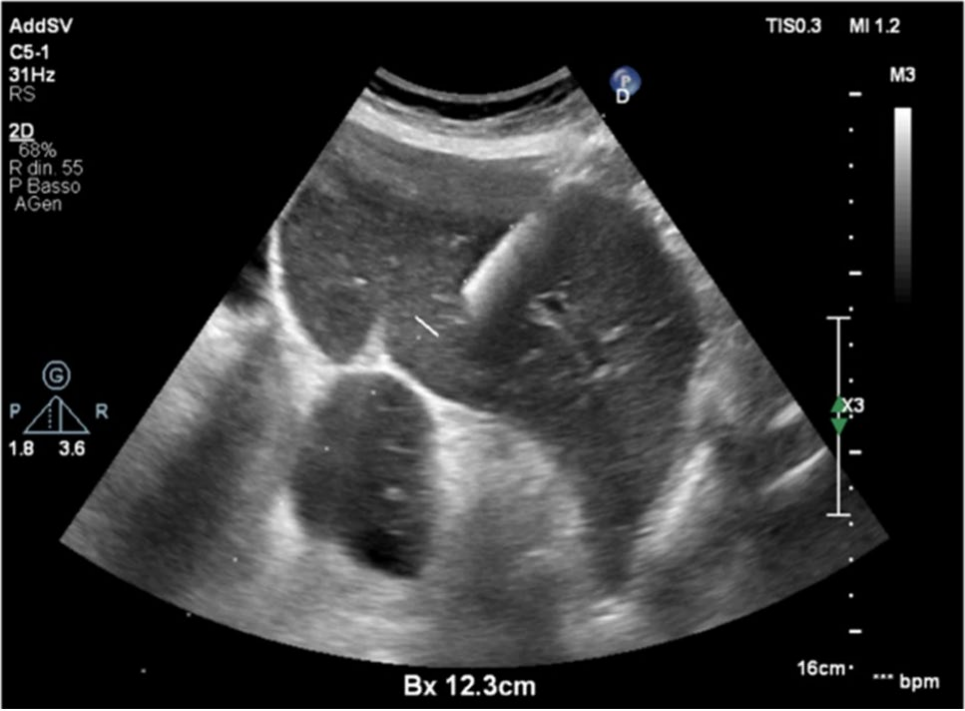
Ultrasound-guided core biopsy on the pathological liver area of the left segments. The procedure was performed by an 18 G semi-automatic needle

**Fig. 5:**
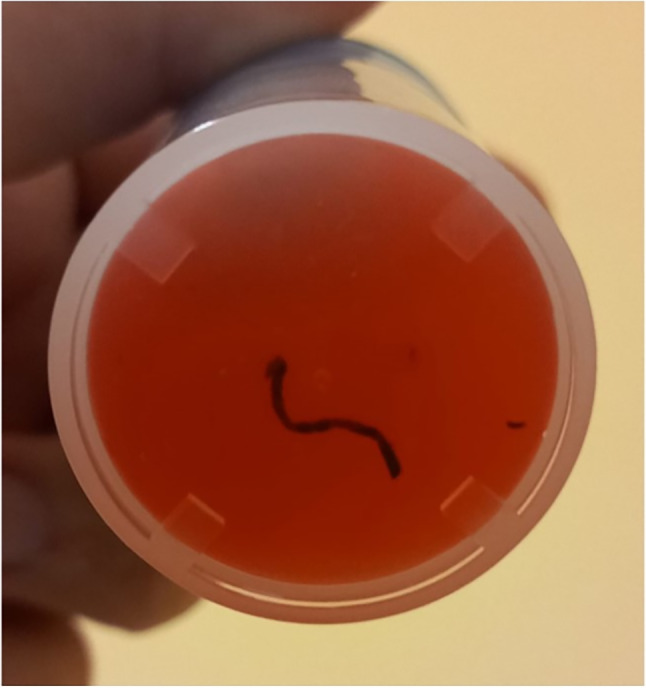
Macroscopic detail of the liver specimen taken through ultrasound-guided biopsy; the dark color of the specimen is evident, which raises the suspicion of tissue of melanocytic origin

On ultrasound examination, the left lobe appeared hypoechoic and slightly heterogeneous, with a sharp demarcation from the right lobe. The left hepatic vein appeared compressed without definite signs of infiltration. CEUS was performed (Fig. 3), thus showing arterial enhancement of the mass in the III–IV liver segments with a prolonged isoechoic phase. Mild washout began in the late phase and became more evident at the fifth minute after the infusion of contrast medium, demonstrating a marked differentiation from the surrounding parenchyma. The lesion was considered of malignant nature by considering the evident, albeit late, washout. An ultrasound guided biopsy of the mass was then performed with a single pass (Fig. 4), thus obtaining a dark colored tissue specimen (Fig. 5).

The histological examination revealed a fragment of pigmented epithelioid neoplasm with morphological characteristics and immunohistochemical profile consistent with a melanoma localization (SOX10 + , focal S100, cytokeratin A1A3).

The patient was subsequently referred to the oncology department and a systemic therapy with Nivolumab was prescribed.

## Discussion and review of literature

Melanoma is characterized by early metastasize in the progression of the disease, and this can occur even from small, thin primary tumours [3]. The skin and subcutaneous tissue are the most common sites for melanoma metastasis; subsequently, metastases may occur in the lungs, liver, bones and brain [4]. A disease-free period can be present following surgical excision of the primary tumour; however, it is quite common to later discover visceral metastases in organs such as the lungs, liver, bones or brain, even months, years or decades after the first treatment. The occurrence of melanoma metastasis is typically related to a poor prognosis, and metastasis are responsible for the most relevant part of morbidity and mortality associated with melanoma [5]. Patients with clinically apparent metastasis to one visceral site display a 1-year survival rate of 36%, while those with metastasis to two different visceral sites have a 1-year survival rate of 13%. For patients with metastasis to three different visceral sites, the 1-year survival rate drops significantly to just 1% [6].

Liver metastases can be clinically detected in 10–20% of patients with metastatic disease from cutaneous melanoma [4]. Sub-clinical metastases to the liver are more common, with estimates ranging from 54 to 77% when examined at the time of autopsy [7–9]. However, while liver is typically not the first site of disease spread in cutaneous melanoma [4], ocular melanoma has been showed to primarily metastasize from the eye to the liver; indeed, liver metastases are detected in approximately 87% of patients with metastatic uveal melanoma [10]. Otherwise, a better prognosis can be observed in patients in whom liver is not involved or when it is not the first site of dissemination [11].

Therefore, liver metastasis can be challenging to identify in early stages, thus being essentially asymptomatic until the disease has advanced. Moreover, liver metastases may occur in unconventional or unexpected ways. A case report showed diffuse liver infiltration by melanoma, where abdominal CT and MRI showed hepatomegaly without focal lesions, a small amount of ascites and a mildly compression of the inferior vena cava due to the presence of the enlarged liver [12]. A percutaneous liver biopsy was then performed, revealing the presence of malignant melanoma cells infiltrating the hepatic sinusoids [12].

Another case demonstrated liver metastasis from melanoma, where it presented as hepatic cysts that rapidly increased in both size and number compared to previous imaging. The magnetic resonance cholangiopancreatography demonstrated several non-enhancing, mildly complex cystic lesions, appearing hypointense on T1-weighted images and hyperintense on T2-weighted images. Fine needle aspiration biopsy showed the presence of numerous malignant cells, some of which had unusual, giant nuclei. Immunostaining confirmed that those cells were related to the primary melanoma [13].

Another rare case of an apparent benign liver lesion hiding a melanoma metastasis was published by Joanna et al. In this case, the patient reported symptoms of fever and pain in the upper right abdomen. A subsequent ultrasound examination revealed the presence of a necrotic mass within the liver; consequently, the initial diagnosis categorized the lesion as a liver abscess. The patient’s medical history was carefully re-examined, and a liver biopsy was performed. Immunohistochemistry revealed that the lesion was consistent with a metastasis originating from a rectal mucosal melanoma, which had been detected during a polypectomy by colonoscopy 1 year prior [14].

Moreover, So et al. reported a clinical case of a liver melanoma metastasis mimicking hepatocellular carcinoma (HCC). The patient had a multinodular liver mass, which was incidentally discovered during a screening ultrasound examination. Multiphase contrast-enhanced CT of the abdomen showed multinodular masses in both hepatic lobes, measuring 6 cm in their largest diameter. The masses displayed typical radiological features of HCC, with arterial phase hyperenhancement and washout during the portal venous and delayed phases. Multiphase MRI of the liver also revealed a contrast enhancement pattern that seemed to confirm the initial suspicion of HCC. Otherwise, an ultrasound-guided percutaneous liver biopsy revealed the presence of malignant cells that were consistent with melanoma [15].

De Toni et al. reported a case of a large liver metastasis originating from choroidal cancer occurring 13 years after diagnosis [16]. In the early arterial phase, the lesion displayed a strong enhancement; in the portal venous phase there was a partial washout of contrast medium from the lesion, and in the delayed phase, 4 min after injection of contrast medium, the lesion showed a hypoechoic, sharply circumscribed enhancement defect [16]. Those contrastographic features are similar to that observed in our clinical case, with a mild wash out well evident only in the late phase.

Furthermore, some works in the literature highlighted how melanoma metastases can be detected in the form of a complex cyst of the liver parenchyma [17] or involve the gallbladder wall [18, 19].

Those clinical case reports highlight the concept that to identify liver metastases from melanoma through imaging can be particularly challenging, thus resulting in diagnostic delays and, subsequently, impacting the prognosis.

## Implications for radiological surveillance

The radiological surveillance in patients with melanoma is an ongoing matter of debate. According to the guidelines from the National Comprehensive Cancer Network (NCCN), patients with stages 0, I, and IIA should undergo a comprehensive physical examination and complete skin examination every 6–12 months for the first 1–2 years, followed by annual check-ups thereafter. Imaging modalities are not recommended for surveillance of stage 0 to IIA except for patients showing signs and symptoms indicative of recurrent metastatic disease. Patients with stage IIB and above are recommended to undergo a comprehensive physical exam and complete skin check every 3–6 months for the first 2 years, followed by every 6–12 months from year 3–5. Imaging considerations, including chest X-ray, computed tomography (CT), positron emission tomography (PET) scans and brain imaging, are suggested every 3–12 months for the first 2 years, then every 6–12 months from year 3–5 for stage IIB and above. Additionally, for patients with a history of brain metastases and those at high risk for brain metastases, such as patients with stage IIIC and above disease, the NCCN recommends more frequent MRI brain scans [20, 21].

In the European Society for Medical Oncology (ESMO) guidelines there is no current consensus on the frequency of patient follow-up and use of imaging; however, they provide general recommendations for monitoring patients at risk for recurrent and new disease. Thin primary melanomas have a small risk of relapse, thus routine imaging is not recommended. In high-risk, US of LNs, CT and/or PET scans are suggested for earlier detection of relapse, although the impact of radiological exams upon survival has not been demonstrated so far. As regards laboratory analysis, serum S100 is recognized as the most accurate marker in the blood for disease recurrence and is used in the follow-up, to monitor disease progression. The ESMO also recommends patient education on avoidance of sunburns, extended unprotected solar or artificial UV exposure, and emphasizes the importance of regular lifelong self-examinations of the skin and peripheral lymph nodes [20, 22].

The British Association of Dermatologists (BAD) follow-up recommendation for in situ melanomas is teaching patients self-examination, with no additional follow-up required. Stage IA melanomas should have medical history and physical examination conducted two to four times over up to 12 months; afterward, they may be discharged from regular follow-up. Patients that have a melanoma in stage IB to IIIA, once they have learned how to self-examine for locoregional metastasis and new primaries, should undergo visits every 3 months for 3 years, followed by 6-monthly visits for up to 5 years; routine investigations are not required. Given the high risk of further metastasis, patients with stage IIIB to C and resected Stage IV melanomas should be visited every 3 months for 3 years from the date of staging, followed by 6-monthly visits for up to 5 years, and then annually for up to 10 years by a Specialist Skin Cancer Multidisciplinary Team; investigations should be conducted based on clinical need and may involve CT surveillance, if considered appropriate. Unresected stage IV melanoma should be visited according to clinical need [21, 23].

The follow-up recommendations in the Guidelines for the Management of Melanoma in Australia and New Zealand (GMMANZ), underline that self-examination in properly trained patients is essential to detect recurrent disease. In addition, patients with stage I disease should undergo physical examination every 6 months for 5 years and yearly thereafter, but more frequent visits should be considered in patients with many atypical naevi, a family history of melanoma, or those who have difficulty in performing self-examination. For patients with stage II and III disease it is recommended to schedule follow-up visits every 3 or 4 months for 5 years, and yearly visits thereafter. There are no specific recommendations for Stage IV disease. Moreover, ultrasound, when performed by experienced ultrasonographers, is a valuable complement to clinical examination in the follow-up assessment of advanced primary disease, although its impact on survival has not been definitively proven [21, 24].

Surveillance should carefully balance the benefits of early diagnosis with a curative treatment option, with the risk of false positives. In this setting, informed consent seems to be a crucial component of the discussion with patients; moreover, the follow-up of any patient with melanoma should always be tailored on the patient’s risk for recurrence [25].

The debate also encompasses the management of surveillance for the less common uveal melanoma. On this topic, a very recent study compared the effectiveness of enhanced surveillance protocols (EP) using high-frequency (liver ultrasound every 3 months) or enhanced modality (incorporation liver computed tomography/magnetic resonance imaging) with the standard protocol (liver ultrasound every 6 months) in detecting metastasis and thus evaluating their impact on the overall survival (OS) of high-risk uveal melanoma (UM) patients. The study revealed that enhanced protocols exhibited a higher rate of smaller metastatic lesions detection when compared to the standard protocol. Specifically, a high-frequency protocol with liver ultrasound every 3 months demonstrated comparable effectiveness to liver imaging through CT/MRI in detecting smaller metastatic lesions, but without improving survival rate [26].

However, currently there is a lack of high-level evidence guiding the optimal approach for monitoring patients’ post-treatment for uveal melanoma [27, 28].

## Implications for metastatic uveal melanoma therapy

Several molecules such as dacarbazine, temozolamide or fotemustine have been used as chemotherapy for metastatic uveal melanoma, but they displayed limited efficacy, with ORRs ranging between 0 and 10% [29–32]. Some recent trials evaluated the MEK inhibitor selumetinib, either alone or in combination with chemotherapy [29, 30, 32]; although some studies demonstrated a significant improvement in PFS, no improvement in OS was observed [29, 32].

The PEMDAC trial tested the efficacy of a combination treatment with pembrolizumab, a monoclonal antibody directed against programmed cell death protein 1 (PD-1), with the histone deacetylase inhibitor entinostat, in order to enhance the expression of immune signalling molecules in melanoma cells. That combination demonstrated a median PFS of 2.1 months and a median OS of 13.4 months [33]. In our case report, it was decided to treat the patient with Nivolumab following oncology consultation. Two other studies evaluated the combination of CTLA-4 and PD-1 inhibition (ipilimumab and nivolumab) thus showing a median OS of 12.7 and 19.1 months, respectively [34, 35]. Eventually, a recent phase III randomized trial evaluated the efficacy of tebentafusp, a bispecific antibody consisting of an affinity-enhanced T-cell receptor fused to an anti-CD3 effector, which redirects T cells to target glycoprotein 100–positive cells [36]. In this work, tebentafusp was related to a significantly longer OS in comparison with treatment with single-agent pembrolizumab, ipilimumab, or dacarbazine (21.7 vs 16.0 months). PFS was lower (median PFS 3.3 months) in the tebentafusp group vs. the control group (2.9 months) [36].

The development of increasingly targeted therapies and the increase of OS imply the setting of a follow-up of patients with melanoma metastases; in particular, quantitative CEUS methods could evaluate the response rate to chemotherapy.

## Conclusions

Our clinical case demonstrates that second-level imaging methods alone may be not enough for diagnosing liver metastasis in a patient with a previous clinical history of uveal melanoma. Contrast-enhanced ultrasonography could support the clinical suspicion of secondary metastasis. Based on our clinical case and the little evidence in the literature, the typical behavior of melanoma metastasis at CEUS would be that of a hyperenhancement in the arterial phase, followed by a mild and late wash-out, thus mimicking the typical behavior of a hepatocarcinoma. Therefore, an ultrasound-guided biopsy seems to be essential to confirm the diagnosis definitively. Therefore, further studies are undoubtedly necessary to evaluate whether liver metastases from melanoma present a peculiar contrastographic features, and to establish an effective melanoma surveillance program, with the hope of achieving improved prognostic outcomes in the future.

## Data Availability

Not applicable.
